# Time to viral load suppression and its associated factors in cohort of patients taking antiretroviral treatment in East Shewa zone, Oromiya, Ethiopia, 2018

**DOI:** 10.1186/s12879-019-4702-z

**Published:** 2019-12-27

**Authors:** Jemal Hassen Ali, Tewodros Getinet Yirtaw

**Affiliations:** Department of Public Health, Ethiopian Field Epidemiology Training Program, St. Paul’s Hospital Millennium Medical College, Addis Ababa, Ethiopia

**Keywords:** Viral load, ART, Ethiopia, Suppression, HIV

## Abstract

**Background:**

A key goal of Antiretroviral Treatment (ART) is to achieve and maintain durable viral suppression. Thus, the most important use of viral load measurement is to monitor the effectiveness of therapy after initiation of ART. The main objective of the study was to determine the time for virological suppression and its associated factors among people living with HIV taking antiretroviral treatments in East Shewa Zone, Oromiya, Ethiopia.

**Methods:**

Patients diagnosed with Human Immunodeficiency Virus presenting to the study health centers between October 3, 2011 and March 1, 2013 were included in the study given the following criteria: age 18 years or greater, eligible to start ART. All patients with baseline viral load measurements were included in the study. Interaction between explanatory variables with the response variable was analyzed by using cross tab features of (Statistical Package for the Social Sciences) SPSS, International Business Machines (IBM) Inc. Significance group comparison was done by Kaplan Meier log-rank test. Cox proportional hazard model was used to select significant factors to the variability between groups.

**Result:**

Plasma viral load was suppressed below the detection level in 72% of individuals taking a different regimen of ART. The median Human Immunodeficiency virus (HIV)-1 plasma viral load in the cohort was estimated to be log 5.3111 copies/ml. The study observed Survival curve difference in the category of marital status (*p*-value 0.023) and baseline cluster of differentiation 4 (CD4) value (*p*-value 0.023). The estimated median time to Plasma Viral Load (PVL) suppression was 181 days (CI: 140.5–221.4) with the age group of 30–39 years having minimum time to achieve suppression with 92 days (CI: 60.1–123.8) and the maximum time required to reach the level was found among the age group between 50 and 59 years.

**Conclusion:**

The study found that the estimated time to achieve PVL after taking ART to be 181 days. Factors affecting time to suppression level were marital status and baseline CD4.

## Background

Human immunodeficiency virus (HIV) is a virus transmitted through different body fluids such as blood and semen. The virus attacks the body’s immune system of which is supposed to protect our body from attack [1]. Acquired immunodeficiency syndrome (AIDS) is a condition caused by the human immunodeficiency virus (HIV) [2].

United nation set goal to ending the AIDS epidemic by 2030 as part of the sustainable development goals (SDG) [3, 4]. Sustainable development goals set target in which: By 2020, 90% of all people living with HIV will know their HIV status, 90% of all people with diagnosed HIV infection will receive sustained antiretroviral therapy and 90% of all people receiving antiretroviral therapy will have viral suppression. When this goal is achieved, it is believed that at least 73% of all people living with HIV worldwide will be virally suppressed. Mathematical modeling suggests that achieving the above mentioned SDG targets by 2020 will enable the world to end the AIDS pandemic by 2030 [5, 6]. Determination of Viral load in the patient plasma is the most important indicator of response to ART. It should be measured in all HIV-infected patient’s plasma at entry into care, at the initiation of ART, and regularly monitoring of progress [7]. In this study, plasma viral load suppression was classified as < 1000 copies/ml as suppressed and plasma viral load of > 1000 copies/ml was classified as an unsuppressed plasma viral load level in the patient plasma. Viral load (HIV-RNA copies/ml of blood plasma) is used as a surrogate marker for disease progression [8]. The main objective of this study was to determine the time to virological suppression and its associated factors among people living with HIV taking antiretroviral treatments in East Shewa zone, Oromiya, Ethiopia, 2018.

## Methods

### Settings

The study was conducted in East Shewa zone Oromiya Region, Ethiopia in 2018. Those patients who take ART treatment in five Health centers in East Shewa zone were included.

The study was conducted on patients who were enrolled between October 3, 2011 and March 1, 2013.

### Study design

Retrospective cohort study design was used to conduct the study.

### Source population

The source population was all people with confirmed HIV positive status in East Shewa Zone, Oromiya region Ethiopia.

### Study population

HIV patients who have started ART service in East Shewa zone between October 3, 2011 and March 1, 2013 with at least two consecutive viral load tests were included.

### Sample size determination and sampling

In this study, 243 study participants who have baseline viral load measurement were included in the study. All patients in the cohort with baseline Plasma viral load were included in the study.

### Data collection tools & procedures

The study used secondary data from a long-running community-recruited prospective cohort of patients living with HIV. HIV Plasma Viral load measurement was taken at enrollment of patient to the study (Baseline), and then after the first month, third month, six months and twelve months and eighteen months of ART treatment. Trained clinicians at the start of the study collected demographic and clinical data, including physical examination details by using structured questionnaires. Interview was also used to collect data from patients. After informed consent, data were collected and checked for completeness.

### Laboratory analysis

All laboratory analyses, except Liquid cultures for Tuberculosis (TB), were performed at Adama Public Health Research and Referral Laboratory Center. CD4 cell counts were analyzed by using BD FACSCalibur cytometer (Becton Dickinson, San Jose, CA). Sputum and Fine Needle Aspirate (FNA) samples were analyzed with direct smear microscopy using Ziehl-Neelsen staining and Xpert MTB/RIF (Cepheid, Sunnyvale, CA) for polymerase chain reaction (PCR). Liquid cultures for TB were performed at International Clinical Laboratories, Addis Ababa, using a BACTEC MGIT 960 (BD Diagnostics, Franklin Lakes, NJ). Plasma HIV-RNA levels were determined using Abbott Real- Time HIV-1 assay (Abbott Molecular Inc., Des Plaines, IL) with a detection limit of 40 copies/ml. External quality assurance of the regional laboratory is regularly performed by the Center for Disease Control and Prevention (Atlanta, GA) [7].

### Eligibility criteria

Human immunodeficiency virus (HIV)- positive patients coming to the study health facilities between October 3, 2011 and March 1, 2013 were eligible for inclusion that met the following criteria: age 18 years or above and residency in the catchment area of any of the study sites. This cohort has since been continuously followed for well over 8 years. Those patients who have started Antiretroviral Treatment (ART) constitute the study population, with follow-up data collected until data abstraction on December 31, 2013 [7].

### Exclusion criteria

Age less than 18 years old, with current or previous ART, as well as patients on ATT for more than 2 weeks before inclusion in this study were excluded from this study.

#### Data quality

Training was given for two data clerks on data management of secondary data obtained from the previous study. Pretest of data collection tools was done before the actual data collection procedure. Double-entry of data made by using EPI INFO 7 to minimize error in data management and analysis was done by SPSS version 21.

### Data analysis

Once data is collected, it was analyzed descriptively to determine the demographic characteristics and distribution of time to PVL. Time to undetectable viral load level was calculated by using Kaplan–Meier to estimate the distribution of PVL in time, and the difference between the survival curves was evaluated with the use of the log-rank test, which is useful for comparing potentially unequal follow-up times of study participants. Cox proportional-hazards regression analysis used to relate covariates with response variable with 95% confidence intervals.

## Result

### Study participant characteristics

Study participants were recruited from Adama, Dhera, Geda, Modjo and Wolenchiti health centers taking ART and selected according to eligibility criteria. The minimum age included in the study was 18 years and the maximum age was 69 years old. Forty-five percent of the participant’s age was between 20 and 29 years old and the minimum category was observed in < 20 years old with 1.6%. The sex distribution of the study participant was 41% male and 59% female participants. Most of the study participants (41%) were married but widowed participants represented only 10% of the participants. At enrollment, 13% of the study participants have a plasma viral load of less than 10,000 copies/μl. plasma viral loads were suppressed in 72% of individuals taking a different regimen of ART treatment. The median HIV-1 plasma viral load in the cohort was log 5.3111 copies/ml. Nine percent of participants’ complete higher education while 36% of them were illiterate. Study participant’s nutritional status was also assessed by the study. Six participants (2.46%) were classified as having acute malnutrition while 71(29.2%) Participants classified as having moderate malnutrition and the rest 166 (68.3%) were classified as normal. The Baseline CD4 count is the value of a CD4 count of the study participants determined before initiating ART. It is determined when the study participant enrolled in the study. Based on the data 140(57.6%) of the participants have Baseline CD4 (BCD4) count of < 200 cells/μl, 77(31.7%) have BCD4 count 200–350 cells/μl and 26(10.7%) have BCD4 count of > 350 cells/μl. Tuberculosis disease screened among participants before enrollment and 189(77.8%) were positive for *Mycobacterium tuberculosis* (MTB) by Acid Fast Bacilli (AFB) light microscopy smearing and Liquide and solid culture Mycobacteria Growth Indicator Tube (MGIT) and Löwenstein–Jensen (LJ) respectively. At enrollment, 77% of study participants without Tuberculosis (TB) disease were found to have BPVL of < 1000 copies/ml, while 84% of participant diagnosed with TB has Baseline Plasma Viral Load (BPVL) of >1000copies/ml. Viral rebound was observed in 16.5% of the study participant. The Proportion of patients having PVL suppressed after taking ART was 72.4%. Study participant contributed 94 person-year of follow up. The incident rate of plasma viral load suppression was 21.8%.

Baseline PVL count in this study is defined as the plasma viral load measured when participant enrolled in the study. At the time of recruitment, 16.6% of female participants and 7% of male participants had BPVL < 1000 copies/μl, While 83.3% of female and 92.9% of male participants’ hade BPVL of >1000copies/μl. The mean and median baseline plasma viral load of study participants were 4.03 × 10^5^cells/μl and 2.03 × 10^5^ cells/μl respectively.

One study participant (0.4%) was put on Stavudine (d4T), Lamivudine (3TC), Nevirapine (NVP) regimen, five study participants (2.1%) were put on Zidovudine (AZT), 3TC, NVP regimen, 13 study participants (5.3%) were put on AZT, 3TC, NVP, 13 study participants (5.3%) was put on AZT, 3TC, Efavirenz (EFV), 17 study participants (7%) were put on Tenofovir Disoproxil Fumarate (TDF), 3TC, NVP regimen and 168 study participants (69.1%) were put on TDF, 3TC, EFV.

### Bivariate analysis

Interaction between explanatory variables with the response variable was analyzed by using cross tab features of SPSS, IBM Inc. The baseline PVL status of a patient did not show significant interaction with the event of interest with χ2 value 2.33(*p*-value .127). Other variables which did not show significance includes age of the patient χ2 value of 4.44(*p*-value .488), Marital status χ2 value of 6.75 (*p*-value .081), occupation χ2 value of .383 (*p*-value .826), education status χ2 value of 1.13 (*p*-value.770), Mid-Upper Arm Circumference (MUAC) χ2 value of .253 (p-value .881), BTB with χ2 value of 1.15 (*p*-value .283). Significant interaction or association with Plasma viral load suppression were observed in sex χ2 value of 5.06 (*p*-value 0.024), Body mass index (BMI) with 0.003, BCD4, χ2 value of 10.98 (*p*-value 0.004) and ART Treatment regimen with χ2 value of 14.23 (*p*-value .0.027).

### Comparison between different categories for survival

Survival distribution for different age groups reveals that there was no significant difference between different age group and other variables in the study in terms of the time to reach suppressed plasma viral load with a log-rank test score of χ2 value 0.860.

The estimated median time to PVL suppression was 181 days (CI: 140.5–221.4) with the age group of 30-39 years having minimum time to achieve suppression with 92 days (CI: 60.1–123.8) and the maximum time required to reach the level was age group between 50 and 59 years.

Significantly different survival distribution curve was observed between different categories of marriage in the study participants. The maximum median time for PVL suppression was observed in unmarried participants with 183 days (CI: 181.7–184.315). The minimum median time registered in the divorced participant category with an estimated median time of 92 days (CI: 91.06–92.93). Significant survival curve difference (log-rank test: χ2 value of 8.84 and *p*-value of 0.012) was observed in HIV patients with different baseline CD4 count. Median survival time for patients with < 200 cells/μl measured 182 days (CI: 160.80–203.19), 181 days (142.39–219.60) for BCD4 200–350 cells/μl, 174 days (CI: 133.40–214.59) and 174 days (CI: 133.40–214.59). No significant difference in Virological Failure was observed between different age groups. Virological failure was observed in 27.6% of the study participants.

### Multivariable analysis

Forward factor selection method (for identifying the synergistic effect of variables on response variables) was used to identify factors that are significantly affected the median time. The Cox-proportional hazard regression method was used to estimate the magnitude of each variable. Variables with a *p*-value of ≤0.05 were included in the model and selected as variables to fit a model that best explains the variance in the equation. Variables identified as significant in this study were marital status with *p*-value 0.023 and baseline CD4 with p-value 0.023. Educational status (*p*-value 0.404), MUAC (*p*-value 0.407), BMI (*p*-value 0.335) and BTB (*p*-value 0.257) were not found to be associated with viral load suppression. The likelihood ratio test of the fit of the full model relative to the intercept only model is statistically significant (χ^2^ = 23.14, *p* = 0.027). This suggests that the model is a significant improvement in model fit relative to the null model. Study participants who are single (SE = 0.318, *p*-value = 0.023) and those study participants with BCD_4_ < 200 cells /μl (SE = 0.0.288, p-value = 0.023) were a significant positive predictor of the hazard of increase in viral load level beyond suppression level.

## Discussion

The median age of study participants in our study was 32 years’ old which is almost similar to the median age of study participants studied by Joseph Davey and et al. in South Africa in 2018 which is 33 years old [9]. The median time for plasma viral load suppression in the study cohort was determined using survival regression analysis. Based on the study, the median time for suppression was 181 days (CI: 140.5–221.4). This finding exactly matches with a study done by the US department of health and human service in 2017, which found the median time between at about 24 weeks after initiation of ART. In another study conducted by Snippenburg W Van, Nellen FJB and Smith C, the median time to undetectable plasma viral load after initiation of treatment was found to be 60 days (12–168 days) which is lower than this study.

As of 2015, we found that 72% of study participants achieved viral suppression, but in a study conducted in Brazil, in which study subjects were followed from May 2000 until July 2001, the percentage of HIV patients achieving viral suppression were 62%. This figure was higher in a study conducted in Zimbabwe in 2018 in which viral load suppression in adult was 87 and 93% in Vietnam in 2016 [10, 11] but similar with the study conducted Cameroon in 2018 which is 72.1% [12] and slightly lower than viral load suppression rate of 76.8% in Study conducted in Cambodia in 2018 [13]. 89% in Uganda studied by Lilian Bulage and et al. in 2017 [14]. This difference could be attributed due to the length of timing of follow up of the patients. A similar study conducted in neighboring Kenya revealed that only 39.85% of patients had virologocal suppression. In another study conducted by Shikuma CM and et al. the percentage of patients taking ART of different regimens reaching virological suppression after taking ART for 16 weeks showed 93% viral suppression. In this study, the percent of patients having virological failure was 16.5%. The same study by Shikuma CM and et al., found that 12.2% of patients have virological failure. Viral suppression rate of this study was found to be similar across different geographical spectrum. Study done in low and middle-income countries of Brazil, Laos people’s democratic republic, Malaysia, Mexico, Myanmar and Republic of Moldova have achieved viral suppression of 80%.

Age category of 30–39 years old had minimum time to viral suppression with 92 days (CI: 60.1–123.8) and the maximum time to achieve viral suppression was found in the age category of 50–59 years. A significant difference in the proportion of different age groups was not found by log-rank test. A study conducted in Kenya by Cherutich found a significant difference between different age categories in achieving viral suppression level. In this study, there was a significant difference between the age group of 15–29 age groups and 30–64 years’ category with a rate of 46.5 and 22.3% respectively.

A significant difference in plasma viral load suppression was observed in the sex category. Pearson’s χ2 for the sex category was 5.06 with a *p*-value of 0.024. A Significant difference in plasma viral load suppression between sex categories was also achieved in a study conducted by Ballesteros-zebad P. in Mexico, which found a high viral suppression rate in male patients compared to female patients. Other studied which found a significant difference between male and female HIV patient’s viral suppression level was done by Pinto M. in southern Brazil, and Gray RH., in Uganda. Viral load suppression across the sex category was not significant in a study done by Rangarajan S. Colby DJ in Vietnam. In this study, which is done in Vietnam, there was no significant difference between male and female HIV patients achieving the undetectable viral level. In this study, viral suppression among women was not significantly different with 93.7% versus 92.9% in males and females respectively.

The median baseline CD4 was similar to a study conducted in Northern Province, Cameron with 204 cells/μl and 192 cells/μl respectively. The Survival curve was not significantly different among different BCD4 categories which is also a result supported by the above study mentioned, but similar study in Cameron by Boelaert M. suggested a significant difference in the survival of patients between different BCD4 category.

Baseline Mid upper arm circumference for evaluation of the patient’s nutritional status does not appear to be associated with a decrease in viral load suppression. Other studies suggested a different association. A study by Liu E, Spiegelman D, found that baseline lower MUAC was associated with a lower rate of viral suppression from patient plasma. Median baseline plasma viral load of study participants was found to be 2.03 × 10^5^ cells/μl which is higher than the study conducted by Patrick Kazooba in Uganda in 2017, which is 8.1 × 10^4^ cells/μl and Colin and et al. in Kenya which is 1.65 10^3^ cells/μl [8, 15].

Body Mass Index affected the survival time of patients with χ2 8.98 and *p*-value of 0.003. BMI category < 18.5 kg/m2 had increased risk of higher viral replication in a study by Sharma A. and Hoover DR, but the association between obesity and reduced or increased viral load was not observed. No difference in survival curve of HIV patients to reach a viral load level for different ART treatment regimens. Snippenburg W. Van achieved the same curve property in a study, which found no difference between protease inhibitor and non-nucleoside reverse transcriptase inhibitor-based regimens.

At least one instance of viral rebound occurred in 16.5% of the cohort of HIV patients in the study period. This figure was 45.1% in the study performed by Guillemi S, Hogg R, Montaner J. equals to the crude incidence of viral rebound of 12.6% compared to 16.4% crude incidence of viral rebound in this study. Variables identified as significant in this study were marital status (*p*-value 0.023) and baseline CD4 (*p*-value 0.023). Pinto et al., found a significant effect of age and ART usage on the survival of patients taking ART drugs.

Educational status (*p*-value 0.404), MUAC (p-value 0.407) BMI (p-value 0.335) and BTB (p-value 0.257) has no significant association. There was also no significant association found in a cohort of patients by sex, marital status, educational level, residence or wealth index. Rangarajan S, Colby DJ Gray RH and et al., found a significantly higher viral load after ART treatment in male and concurrent infections with Tuberculosis, Herpes virus, malaria and helmenthiasis. A Study done by Semu H. and Hawkin C. also confirms that baseline MUAC and plasma viral load level does not have significant relation.

## Conclusion

The estimated time to achieve PVL after taking ART was found to be 181 days. Factors affecting time to suppression level were marital status and baseline CD4. Additional factors believed to be influencing the survival curve were also studied, but no significant relation was found with the response variable.

## Data Availability

This cohort (longitudinal) study is ongoing starting from 2011 GC until now; I cannot deposit the data in the public domain. Data is available at corresponding author upon request. Additional data is available at Lund University, Sweden, contact person, Dr. Anton Reepalu (anton.reepalu@med.lu.se).

## Abbreviations

3TC: Lamivudine
AFB: Acid fast bacilli
AIDS: Acquired immunodeficiency syndrome
ART: Antiretroviral treatment
BCD4: Baseline cluster of differentiation 4
BMI: Body mass index
BPVL: Baseline plasma viral load
BTB: Baseline tuberculosis
CD4: Cluster of differentiation 4
d4T: Stavudine
EFV: Efavirenz
FNA: Fine needle aspirate
HIV: Human immunodeficiency virus
IBM: International business machines
LJ: Löwenstein–Jensen
MGIT: Mycobacteria growth indicator tube
MTB: *Mycobacterium tuberculosis*
MUAC: Mid-upper arm circumference
NVP: Nevirapine
PCR: Polymerase chain reaction
PVL: Plasma viral load
SDG: Sustainable development goals
SPSS: Statistical package for the social sciences
TB: Tuberculosis
TDF: Tenofovir disoproxil fumarate
